# From evidence to impact: purposeful use of implementation science theories, models, and frameworks in genomics and precision health

**DOI:** 10.3389/fpubh.2026.1949272

**Published:** 2026-10-30

**Authors:** Soroush Fariman, Stephanie Best, Natalie Taylor, Megan C. Roberts

**Affiliations:** 1 Division of Pharmaceutical Outcomes and Policy, Eshelman School of Pharmacy, University of North Carolina at Chapel Hill, Chapel Hill, NC, United States; 2 Melbourne School of Health Sciences, University of Melbourne, Melbourne, VIC, Australia; 3 Implementation to Impact, University of New South Wales, Sydney, NSW, Australia

**Keywords:** genomic medicine, health services research, implementation science, knowledge translation, precision health, theories, models, frameworks

## Abstract

A growing number of genomics precision health applications are supported by robust evidence, yet their integration into routine clinical and population health practice remains slow, uneven, and inequitable. This article demonstrates how implementation science (IS) theories, models, and frameworks (TMFs) can support the translation of evidence-based genomics applications by presenting three cases from pediatric precision oncology, reproductive genetic carrier screening, and population genomic screening for hereditary conditions. Across the three cases, IS TMFs were purposefully selected, adapted, combined, or supplemented to fit the implementation context, core complexities, and the conceptual work required, and were revisited as implementation evolved. We argue that this purposeful approach can provide an operational backbone for integrating precision health innovations into real-world systems of care and help transform individual implementation efforts into transferable knowledge.

## 1 Introduction

Among the many precision health innovations, genomics applications stand out as some of the most mature and increasingly ready for widespread implementation (1). Landmark initiatives such as the Human Genome Project (2) contributed to the rapid expansion of our genomic knowledge and enabled the development of lower-cost sequencing-based technologies, leading to a growing array of genomics precision health innovations (3). While evidence and implementation readiness vary, several genomics applications in clinical and population health, including genomics-guided oncology treatments (4), reproductive genetic carrier screening for prospective parents (5, 6), and population-based screening for certain hereditary conditions (7), are now supported by robust evidence and poised for implementation at scale, placing the promise of precision health increasingly within reach.

Despite the promise, translating evidence-based precision health innovations into routine clinical or population health practice remains challenging. For genomics applications, successful implementation often involves redesigning clinical workflows and care processes, strengthening information technology and laboratory infrastructure, building workforce capacity, and aligning reimbursement models and incentives, requiring changes across individual, organizational, health system, and policy levels (8). In addition, unique concerns regarding genetic privacy and discrimination (9, 10) with implications that extend beyond the individual to their biological family and relatives (11) further complicate implementation. Traditional approaches to planning and evaluation often struggle to capture these interacting elements and how they evolve over time. Not surprisingly, despite many system- or national-level initiatives to implement evidence-based genomics applications in real-world practice (12), uptake has remained slow and uneven (13). Moreover, their benefits have not been equitably realized, in part because of the underrepresentation of diverse populations in genomics research and evidence (14, 15). Genomics now faces a translational bottleneck hindering the efficient, equitable, and scalable realization of its potential to improve patient and population health.

To systematically address these challenges, experts have called for implementation science (IS) to provide the foundation for the translation of evidence-based genomics applications into clinical and population health practice (16–18). IS offers a broad range of theories, models, and frameworks (TMFs) to guide this work: Theories are explanatory and specify relationships among constructs to describe how or why particular outcomes occur; models typically provide simplified representations of processes or phenomena; and frameworks organize constructs to structure understanding of a phenomenon. Nilsen (19), in their seminal paper, further classified TMFs by function as determinant frameworks, which identify barriers and facilitators; implementation and classic theories, which explain mechanisms; process models, which guide implementation; and evaluation frameworks, which assess implementation success or failure. While these functions are complementary, the expanding number of TMFs can make it challenging to select approach(es) that best fit a particular implementation problem.

The use of IS TMFs in genomics was historically limited (20–22), but has increased in recent years (23), in part driven by funding priorities emphasizing real-world implementation (24). Despite additional work associated with using IS TMFs to guide implementation efforts, prior theory-based implementation studies have demonstrated improvements in clinical and cost-effectiveness outcomes, supporting their potential to facilitate more efficient and timely translation of evidence-based interventions into practice (25, 26). To illustrate the purposeful use of IS TMFs in genomics implementation, we present three cases spanning different contexts and stages of translation. We show how TMF use was tailored to the implementation problem and conceptual work required and revisited as implementation evolved.

## 2 Case examples of IS TMF use in genomics implementation

### 2.1 Genomics service innovation supporting national access to precision-guided therapies in pediatric oncology

In pediatric precision oncology, clinicians must interpret evolving genomic data, identify appropriate precision-guided therapies, and navigate fragmented pathways to access these therapies across institutional and jurisdictional boundaries. An ecosystem of service-level interventions (e.g., data platforms, molecular tumor boards, and decision support systems) is needed to support the implementation of the clinical intervention (i.e., precision-guided treatment). These interdependencies can obscure how clinical decisions are made in practice, how supporting platforms and processes influence them, and ultimately how implementation strategies shape access to genomics-guided care (27–29). ProCure, a medicines database designed to support clinicians in identifying and accessing precision-guided therapies, is one such service-level intervention. ProCure operates alongside other service-level interventions, such as molecular tumor boards and decision-support systems, to facilitate clinical decision-making (30), both supporting and shaping clinicians’ ability to identify and access precision-guided therapies (31).

Although IS offers a suite of complementary TMFs for examining implementation complexities and addressing them through targeted strategies, they are often applied in isolation, making it difficult to link determinants, strategies, mechanisms, and outcomes within a coherent analytic structure, particularly when multiple clinical and service-level interventions are in play (32–36). Implementation Research Logic Models (IRLMs) have emerged as a practical tool to integrate these key components within a single coherent framework (37). Building on this foundation, we used an adapted Clinical, Service, and Implementation Intervention - Implementation Research Logic Model (CSII-RLM) to better reflect the realities of precision health implementation by incorporating multiple interacting layers of intervention, including clinical, service, and implementation components (31).

To operationalize this approach, implementation determinants identified through the Consolidated Framework for Implementation Research (CFIR) (38, 39) and the Theoretical Domains Framework (TDF) (40, 41) informed strategies at both the clinical and service levels. The Expert Recommendations for Implementing Change (ERIC) (42) and Behavior Change Techniques (BCTs) (43) supported specification of strategies and their active ingredients, which were situated within the CSII-RLM. All components were then aligned within the CSII-RLM to link determinants, strategies, mechanisms, and outcomes, including implementation outcomes (e.g., acceptability, appropriateness, feasibility) and downstream service and clinical outcomes (e.g., timely identification and access to precision-guided therapies). Critically, this layered approach makes explicit that precision medicine (as a clinical intervention) is enabled (and possibly sometimes constrained) by service-level interventions that require implementation in their own right. By representing these layers within a single model, the CSII-RLM captures how tools such as data platforms, molecular tumor boards, and decision-support systems both support and shape clinical decision-making, giving rise to dynamic and nonlinear implementation processes.

The use of an integrated, layered CSII-RLM provided several key benefits. First, it enhanced conceptual clarity by distinguishing how the service intervention (ProCure) supports the clinical intervention (precision-guided treatment) and making explicit how determinants, strategies, mechanisms, and outcomes connect across layers. Second, it supported more precise specification of strategies by linking them to theory-informed mechanisms of action. Third, the CSII-RLM made this pathway explicit across clinical, service, and implementation layers, linking implementation outcomes such as acceptability and feasibility to downstream service and clinical outcomes such as timely access to precision-guided therapies. For example, resource-intensive application processes and time-sensitive decision-making (barriers to accessing precision-guided therapies) were addressed through ProCure’s centralized drug evidence, access information, and examples of successful applications, thereby reducing information and process burden (hypothesized mechanism) and facilitating clinicians’ ability to identify and pursue precision-guided treatment opportunities (proximal outcome). Importantly, the CSII-RLM also supported iterative refinement over time by providing a structure for revisiting hypothesized pathways and adapting strategies as implementation progresses. By structuring data collection and analysis around hypothesized pathways, the model enabled testing and adaptation of strategies in response to emerging findings, strengthening both internal validity and external relevance.

### 2.2 Testing the feasibility and adoption of a national reproductive genetic carrier screening program

Interest in reproductive genetic carrier screening (RGCS) is increasing as awareness grows that many couples without a family history may unknowingly be carriers of genetic conditions. Historically, carrier screening was limited to condition-specific tests (e.g., Tay-Sachs or cystic fibrosis), often guided by ancestry or family history. Recent advances have made it feasible and increasingly affordable to screen for multiple conditions with a single test (44). RGCS can identify prospective parents with an increased likelihood of passing on an inherited condition and support informed decision-making about reproductive options, such as *in vitro* fertilization with preimplantation genetic testing or prenatal testing (45).

Professional bodies now recommend that antenatal care providers, including obstetricians, fertility specialists, and other relevant providers, offer RGCS to prospective parents (46). However, uptake varies substantially among providers. While obstetricians may be more likely to discuss RGCS with their patients, general practitioners, who play a critical role in preconception and antenatal care, report barriers such as limited confidence, variable interest, time pressures, and concerns about medicalizing pregnancy. Addressing these complexities calls for implementation strategies that take context, content, and processes into account and involve frontline providers in the design and evaluation of RGCS delivery models (47).

The Australian Reproductive Genetic Carrier Screening Project, also known as Mackenzie’s Mission, aimed to inform a government-funded program covering at least 1,281 genes and over 750 autosomal recessive and X-linked conditions for more than 10,000 couples (5). We drew on IS, first, to investigate challenges faced by frontline providers in delivering RGCS, co-design implementation strategies to support its adoption, and evaluate their impact; and second, to identify the conditions needed to scale and sustain a national RGCS program over time.

In the first step, informed by the Theoretical Domains Framework (40, 41), we investigated providers’ intuitive and empirical implementation challenges through a systematic review and subsequent exploratory interviews. Theory-informed behavior change techniques were then linked to the highest-scoring domains. For instance, challenges related to ‘skills’ (e.g., limited practice in offering RGCS) were linked to ‘instructions on how to perform behavior’, and those related to ‘environmental context and resources’ (e.g., lack of time) were linked to ‘prompts/cues.’ We then co-designed implementation strategies and trialed a skills video and waiting room poster (6).

In the second step, we used CFIR (38, 39) to investigate factors influencing program scale-up and sustainment at the national level. Data were collected from ‘implementation science questions’ posed at each program committee meeting, e.g., National Steering, Variant Review, and National Operational committees, as well as from interviews with study team members. We linked findings to the Expert Recommendations for Implementing Change (42) to generate implementation strategies across the implementation timeline, from early (e.g., design quality and packaging) to future (e.g., cosmopolitanism) (48). The trialed implementation strategies were tested and showed a reduction in barriers related to both ‘skills’ and ‘environmental context and resources.’ These findings informed future implementation of RGCS, particularly stressing the need for support beyond education in isolation. The CFIR further highlighted the importance of networks and communications, particularly clinicians’ access to expertise and knowledge, and the need to clearly articulate the relative advantage of RGCS to clinicians to support scale-up and sustainability.

### 2.3 Pilot implementation of population genomic screening in a large health system

Millions of people in the US carry pathogenic genetic variants that confer substantially elevated risks of serious but preventable diseases such as Lynch syndrome (LS), Hereditary Breast and Ovarian Cancer (HBOC), and Familial Hypercholesterolemia (FH). DNA-based screening of healthy individuals has an enormous yet untapped potential to improve outcomes through prevention and early detection before symptoms manifest. Though the promise of genomic screening to transform precision population health is widely acknowledged, integration into routine practice remains limited.

In 2018, the National Academies of Sciences, Engineering and Medicine’s Genomics and Public Health Action Collaborative provided a roadmap for expanding risk-based genomics programs to pilot population screening programs (49). Importantly, the report emphasized a critical need to address implementation. Beginning June 2021, the University of North Carolina (UNC) Program for Precision Medicine in Healthcare piloted a clinical offering for genetics-based screening for the multiple genes associated with LS, HBOC and FH, called the UNC Precision Health Genetic Screening (PHGS) (50). IS TMFs were used to understand key barriers and facilitators of the program and develop strategies to address these determinants and refine the program.

First, CFIR (38, 39) was used to examine multilevel barriers and facilitators to PHGS implementation. A systematic review of the literature, informed by CFIR, identified limited prior work on clinic- and policy-level barriers to implementing population genomic screening. We prioritized constructs from ‘Inner and Outer Settings’ to conduct qualitative interviews examining key barriers and facilitators from the implementation team perspective (e.g., genetic counselor, project manager, community engagement expert, genetics educator). A second interview guide was developed to examine implementation challenges from participants’ perspectives, this time emphasizing constructs related to ‘Characteristics of Individuals.’ To better capture individual-level health behavior constructs not specified in CFIR, such as perceived severity of the conditions screened for, we supplemented it with the Integrated Behavioral Model (51).

These findings served as the basis for developing responsive strategies for refining the PHGS program. We used implementation mapping, a five-step process used to develop implementation strategies that are aligned with identified barriers (52). For example, we identified a policy-level barrier affecting the health system’s ability to offer PHGS at no cost to Medicare and Medicaid beneficiaries, due to the Beneficiary Inducement Clause, a federal law intended to prevent “patient steering” (53), but we also learned from participants that free screening was a major enabler for their participation in PHGS. We also found a need to increase primary care provider buy-in, both from our implementation team and participants, to better integrate PHGS into routine workflow. Following a priority-setting session with our implementation team, we used a matching tool to align the key CFIR constructs with implementation strategies and prioritized two broad strategies for the future program refinement: (i) identifying alternative funding mechanisms to increase access to PHGS, and (ii) engaging primary care providers to support integration into routine primary care. These strategies will inform the next phase of PHGS implementation and evaluation.

## 3 Discussion

Across our cases, we illustrate the value and complexities of using IS TMFs to support the translation of three evidence-based genomics applications into clinical and population health practice. Our cases spanned different applications, contexts, and stages of implementation, each faced distinct complexities, and called for different conceptual work to operationalize the intervention within systems of care. Beyond providing a common vocabulary, IS TMFs helped structure understanding and planning for real-world implementation, diagnose challenges, link determinants to tailored strategies, attend to multiple levels of change and their interactions, and define implementation outcomes alongside clinical and service outcomes. Table 1 summarizes the process.

**Table 1:** Purposeful application of implementation science theories, models, and frameworks (TMFs) across three genomics implementation cases.

TMF application process		Case 1: Pediatric precision oncology (ProCure)	Case 2: Reproductive genetic carrier screening (Mackenzie’s Mission)	Case 3: Population genomic screening (UNC PHGS)
**Implementation context**		Developing a service innovation to support national access to precision-guided therapies for children with cancer in Australia	Implementing a national reproductive genetic carrier screening program for couples planning or in early pregnancy in Australia	Piloting clinical offering for population genomic screening for CDC Tier 1 conditions within an academic U.S. health system
**Core implementation problem/challenge**		Complex service ecosystem for identifying and accessing genomics-guided therapies	Evolving from frontline provider adoption to national scale-up and sustainment	At the time, little was known about multilevel implementation determinants across policy, health system and workflow, provider, and participant levels
**TMF role/function(s)**		Connect clinical, service, and implementation layers	Address implementation problems as the program moved from adoption to scale-up and sustainment	Provide multilevel breadth while adding behavioral specificity
**Select/combine/adapt TMFs**	To identify determinants	CFIR + TDF identified barriers (e.g., time-sensitive decisions) and facilitators (e.g., multidisciplinary care)	TDF identified early provider-level barriers (e.g., skills and environmental context/resources). CFIR later identified determinants of scale-up and sustainment (e.g., networks/communication, access to expertise, and relative advantage)	CFIR provided a multilevel determinant scaffold; the Integrated Behavioral Model supplemented CFIR for participant-level behavioral constructs (e.g., financing/access constraints, PCP buy-in/workflow integration, and participant beliefs)
	To design/tailor strategies	Determinants/end-user needs informed co-design of ProCure (centralized drug evidence, access information, application examples); ERIC + BCTs specified implementation strategies	TDF domains were linked to BCTs and co-designed strategies, including a skills video and waiting-room poster. Later, CFIR findings were linked to ERIC to inform strategies for scale-up and sustainment	Implementation Mapping and CFIR-ERIC Matching Tool identified priorities (alternative funding mechanisms to increase access and PCP engagement to support integration into routine primary care)
	To specify mechanisms and outcomes	Adapted CSII-RLM linked determinants and strategies to hypothesized mechanisms and implementation, service, and clinical outcomes (Example: resource-intensive access process → centralized ProCure drug/access information → reduced information and process burden → improved acceptability/appropriateness and timely access to precision-guided therapies)	Trialed strategies reduced barriers related to skills and environmental context/resources; later work extended attention to scale-up and sustainment	To be tested in future work: Financing constraint → alternative funding → increased access; PCP buy-in/workflow barrier → PCP engagement → routine-care integration
**Lessons learned**		Combine TMFs when needed to connect interacting intervention layers and make hypothesized causal pathways explicit	Reassess TMF fit as the implementation problem changes across the implementation lifecycle	Supplement broad multilevel frameworks with complementary theories for more explanatory depth at different ecological levels

BCTs, behavior change techniques; CFIR, Consolidated Framework for Implementation Research; ERIC, Expert Recommendations for Implementing Change; IS, implementation science; PCP, primary care provider; TDF, Theoretical Domains Framework; TMFs, theories, models, and frameworks; IBM, Integrated Behavioral Model; PHGS, Population Health Genomics Screening; CSII-RLM, Clinical, Service, and Implementation Intervention Research Logic Model.

Prior studies showed limited use of IS TMFs in genomics implementation (20–22). As the field has evolved, the central question is shifting from *whether* TMFs are used to *how* purposefully and coherently they are applied to inform implementation efforts and generate transferable learning (54, 55). Purposeful use begins with selecting the TMF(s) that best fit the implementation context and problem and, where needed, adapting, combining, or supplementing them to address the conceptual work required. This can be challenging, and if poorly executed, may lead to poor fit or superficial use (e.g., using constructs primarily to catalogue barriers and facilitators without informing implementation decisions), and missed opportunities to generate implementation knowledge (56–60). This is not unique to genomics. In addition to numerous available TMFs, selection is often complicated by the context-dependent, multilevel, and dynamic nature of implementation, such that no single TMF may provide sufficient conceptual coverage or specificity to address every implementation question. In such cases, purposefully combining or supplementing existing TMFs can provide complementary explanatory or operational value while retaining links to broader IS knowledge. This is particularly relevant for genomics, given the nested service infrastructure, rapidly evolving evidence, distributed expertise, policy and reimbursement constraints, and close coupling between implementation and equitable access. We highlighted this purposeful approach to TMF selection and adaptation across our three cases, in which the implementation context and core challenge determined the conceptual work and, in turn, the selection, combination, supplementation, or adaptation of TMFs. More specifically, in ProCure (Case 1), the complexity of supporting precision-guided oncology treatment within an ecosystem of interacting clinical and service interventions, called for TMFs to function as a causal architecture, making explicit and connecting determinants, strategies, mechanisms, and outcomes across layers. In Mackenzie’s Mission (Case 2), implementation priorities shifted from provider adoption to national scale-up and sustainment, and TMF use evolved responsively to support each stage. In UNC-PHGS (Case 3), multilevel complexity required TMFs to function as a pragmatic diagnostic and design system, using complementary theories and methods to identify determinants and tailor strategies across levels. In each case, TMF use evolved *adaptively* across the implementation lifecycle in response to evolving priorities, and when needed, IS TMFs were complemented by other IS tools (e.g., determinant–strategy matching tools (61)), techniques (e.g., Behavior Change Techniques (43)), or non-IS TMFs (e.g., behavioral theories such as the Integrated Behavioral Model (51)). Such combinations should serve a clearly defined and complementary function or address an identified conceptual gap (62, 63). Where appropriate, building on established TMFs may support greater comparability and cumulative learning than developing new frameworks (56, 59). This purposeful approach, summarized in Table 1, can serve as a practical guide for genomics implementation researchers and practitioners seeking to apply IS TMFs in their own work.

Beyond these cases, IS TMFs have been applied in a wide range of precision health initiatives, including efforts to develop genomic-specific frameworks and adapt or extend existing IS approaches for genomics research and practice (64, 65). As more evidence-based genomics applications are incorporated into clinical and population health practice, there is an opportunity to move from using TMFs to purposefully orchestrating them to support implementation while also generating systematic learning. Emerging guidance from the broader IS field can help genomics researchers select TMFs based on their purpose, attributes, and fit with the implementation context (54, 59, 66). To make these theoretical choices empirically useful, a warranted next step for IS in genomics is to clearly define and measure relevant constructs, specify how implementation strategies are expected to work, and test whether hypothesized mechanisms hold in practice (67). For genomics, with rapidly evolving evidence, technologies, infrastructures, workforces, and policy environments, this learning should also inform scale-up, sustainability, and equity.

Of particular relevance to genomics and precision health, IS TMFs can also provide a shared structure for transdisciplinary collaboration by organizing different stakeholders’ perspectives (e.g., clinicians, genetic professionals, and implementation teams to patient participants and other program partners) around common implementation issues. Without shared concepts, standardized terminology, and theory-informed approaches, lessons from growing precision health implementation efforts will remain difficult to compare and transfer across settings. Although structured implementation work takes effort, it can help ensure that changes to service delivery, clinical workflows, and engagement with patients, communities, and other implementation partners are systematic, context-responsive, and grounded in evidence. We encourage others who aim to operationalize precision health in practice to make use of IS TMFs purposefully; done well, such work can turn individual projects into cumulative, transferable learning that brings both IS and precision medicine closer to fulfilling their promise (18).

## Data Availability

The original contributions presented in the study are included in the article/supplementary material, further inquiries can be directed to the corresponding author.
